# Three-Dimensional Measuring Device and Method of Underground Displacement Based on Double Mutual Inductance Voltage Contour Method

**DOI:** 10.3390/s22051725

**Published:** 2022-02-23

**Authors:** Nanying Shentu, Feng Wang, Qing Li, Guohua Qiu, Renyuan Tong, Siguang An

**Affiliations:** 1 College of Mechanical and Electrical Engineering, China Jiliang University, Hangzhou 310018, China; stnying@cjlu.edu.cn (N.S.); wfcjlu@163.com (F.W.); tongrenyuan@126.com (R.T.); 2 College of Information Engineering, China Jiliang University, Hangzhou 310018, China; qghfr@163.com (G.Q.); annsg@163.com (S.A.)

**Keywords:** underground displacement monitoring of landslide, three-dimensional measurement, double mutual inductance, voltage contour method

## Abstract

Landslide is a very common and destructive geo-hazard, and displacement monitoring of it is integral for risk assessment and engineering prevention. Given the shortcomings of current landslide displacement monitor technologies, a new three-dimensional underground displacement monitoring technology is proposed based on the double mutual inductance voltage contour method. The underground displacement measuring device mainly consists of an information processing unit and sensing array, connected by power and RS-485 communication lines. An underground displacement measurement model to convert the double mutual inductance voltages and the inter-axis angle into the relative displacement between adjacent sensing units is established based on the interval-interpolation and contour-modeling. Under the control of the information processing unit, the relative displacement between any two adjacent sensing units can be calculated through the underground displacement measurement model, so as to obtain the total displacement from underground depth to surface, and the measurement data can be further sent to the Internet of things cloud platform through the 4G module; thus the remote real-time monitoring of underground displacement three-dimensional measurement for the rock and soil mass from underground depth to the surface is realized. The measurement model is verified by building an experimental platform to simulate the underground displacement of rock and soil mass. The experimental results show that for each measuring unit, when the horizontal displacement and vertical displacement are within the measurement range of 0–50 mm, the maximum measurement error will not exceed 1 mm, which can meet the accuracy requirements of underground displacement monitoring of landslide.

## 1. Introduction

Landslide hazard is the most common geological hazard in nature and causes billions of dollars of damage every year worldwide. China is one of the countries most seriously affected by landslide hazards in the world [1]. According to the statistics of the Ministry of Natural Resources of the People’s Republic of China, the number of landslide hazards and the caused losses each year in China are the highest among all geological hazards, seriously threatening public health and safety. To deeply study the evolution process of landslide and master its motion law, significantly reduce or prevent the loss caused by landslide hazard and timely ensure the safety of people’s lives and property, precise and continuous monitoring of landslide-prone areas is required [2].

There are several components of landslide monitoring, including displacement, strain, hydrology, precipitation, ground temperature, and other environmental factors [3]. Among them, displacement is the most direct state quantity reflecting landslides, so that monitoring landslide displacement can play a more accurate role in early warning and forecast [4]. Nowadays, displacement monitoring of landslides has been proven to be the most cost-effective risk reduction measure [5,6,7]. Scientists worldwide have put forward a variety of methods for landslide displacement monitoring, which can be divided into two main categories: surface displacement monitoring and underground displacement monitoring [8].

Total station, interferometric synthetic aperture radar (InSAR), global navigation satellite system (GNSS), and depth camera are widely used in surface displacement monitoring [9,10,11,12,13]. The total station is easy to operate and can automatically locate the preset measuring points. Manconi et al. [14] used a total station to continuously monitor surface displacements for up to one year after the earthquake in the village area of Paganica, central Italy. However, its measurement range is relatively small and more suitable for small and medium-sized landslide monitoring and requires ground intervisibility (observation cannot be conducted on rainy and foggy days). InSAR has wide coverage and high spatial resolution. Karimzadeh et al. [15] investigated the long-term surface displacement in East Azerbaijan province of Iran by InSAR. However, the correlation of its interference images depends on seasonal and weather conditions. GNSS has high measurement accuracy, and the continuous measurement accuracy can reach the millimeter level. Notti et al. [16] used GNSS to monitor the unstable slope affecting Madonna Del Sasso Sanctuary. However, its power consumption is large and large-scale solar panels and batteries are usually required in fieldwork, so GNSS are generally used in large landslide areas. A depth camera is a new technology that has emerged in recent years, and its cost is relatively low compared with other surface displacement monitoring technologies. Caviedes-Voullième et al. [17,18] proposed to use RGB-D sensors to measure the surface of dry granular flows. Nichols et al. [19] proposed to use Kinect (a consumer-grade RGB-D sensor released by Microsoft) to measure the surface of gravity waves in clear water. Although one single device cannot monitor large scales, multiple devices can be connected and used to monitor larger domains.

The above techniques can measure local and regional displacement at the surface, but surface displacement is often hysteretic and susceptible to topographic and environmental factors. By comparison, underground displacements can more accurately analyze the internal deformation characteristics of rock and soil mass, understand the landslide’s real evolution, and obtain the local deformation of the entire landslide body. Therefore, under-ground displacement monitoring is more effective than surface displacement monitoring in predicting the trend of landslides [20].

Borehole inclinometer [21,22], time-domain reflectometry (TDR) [23,24] and optical fiber sensing technology [25] are widely used in underground displacement monitoring. A borehole inclinometer is an in-situ monitoring instrument that derives horizontal displacement by measuring the borehole tilt angle. It is a widely accepted monitoring technology with a relatively simple structure [26]. According to its installation mode, it can be divided into a fixed inclinometer and a sliding inclinometer. The fixed inclinometer embeds the inclinometer probe directly on the internal measuring points of the structure and is generally used for high and steep slopes with a complex geographical environment. In order to realize the continuous measurement of landslides, Ruzza et al. [27] developed a multi-module fixed inclinometer. The sliding inclinometer needs to pre-bury the inclinometer tube and drive the inclinometer probe with a guide sliding wheel to detect section by section in the inclinometer tube. Zhang et al. [20] proposed a landslide displacement measurement method based on deformation-coupled pipeline trajectory measurement, which has been successfully applied to the deformation monitoring of the Majiagou landslide. Fixed inclinometer and sliding inclinometer have advantages and disadvantages; the former is more costly but can realize automatic measurement. The latter is less costly but less efficient in monitoring: each measurement requires manual operation, which has a great hidden danger to the safety of construction personnel. Meanwhile, both are unfit for large deformation monitoring. TDR technology embeds the coaxial cable into the rock, and soil mass uses the coaxial cable to couple the landslide deformation and analyzes the coaxial cable’s deformation position and deformation degree by measuring the return time and vibration amplitude of the reflected signal. Chung et al. [28] successfully captured the sliding surface with TDR technology. However, this method can only determine the shear plane and it is difficult to determine the displacement size and displacement direction. The coaxial cable is easy to break under the condition of large deformation [29,30]. Optical fiber sensing technology has the advantages of waterproof, corrosion-resistance, and anti-electromagnetic interference, so it is widely used in many fields of landslide hazard monitoring [31,32,33]. According to the different sensing principles, it can be divided into fiber Bragg grating (FBG) and Brillouin optical time-domain reflection (BOTDR) sensing technologies. FBG sensing technology is mainly used to reflect external deformation information by detecting changes in the central wavelength of the sensor, which can be used to measure temperature, strain, displacement and pressure [34,35]. The bare FBG sensor is fragile and usually needs to be fixed on a specific carrier [36,37,38]. Wang et al. [4] proposed a detection device combining FBG and PVC pipe, Xu et al. [39] proposed a soft FBG strain sensor composed of rubber strip, FBG, and steel plate. This method reflects landslide deformation through carrier deformation, so the fixed carrier often affects it. BOTDR sensing technology reflects the deformation information by detecting the influence of fibre-optical deformation on the reflected light frequency. Gage et al. [40] had been successfully used BOTDR to monitor strain in rock and soil mass, but the technology has a low spatial resolution poor real-time [41,42]. Both technologies have a limited measurement range, and have the same risk of fracture under large deformation conditions.

In general, although the above underground displacement monitoring methods can monitor the underground displacement of landslides, they all have their shortcomings in one way or another. They have a standard limitation: unable to watch the three-dimensional changes of underground displacement. However, only the three-dimensional changes of underground displacement can truly characterize the movement trend in the rock and soil mass to achieve the purpose of early warning and forecasting. In order to solve the limitations of the existing underground displacement monitoring technology, we designed an integrated underground displacement three-dimensional measuring sensor based on double mutual inductance voltage contour method, applied the Internet of Things technology to this system, and finally realized a highly automated and high-precision remote monitoring system of three-dimensional measurement of underground displacement. It solves the limitations of the current underground displacement measurement technology and has great research significance and practical value.

## 2. Structural Design and Working Principle

### 2.1. System Structure Design

**Figure 1:**
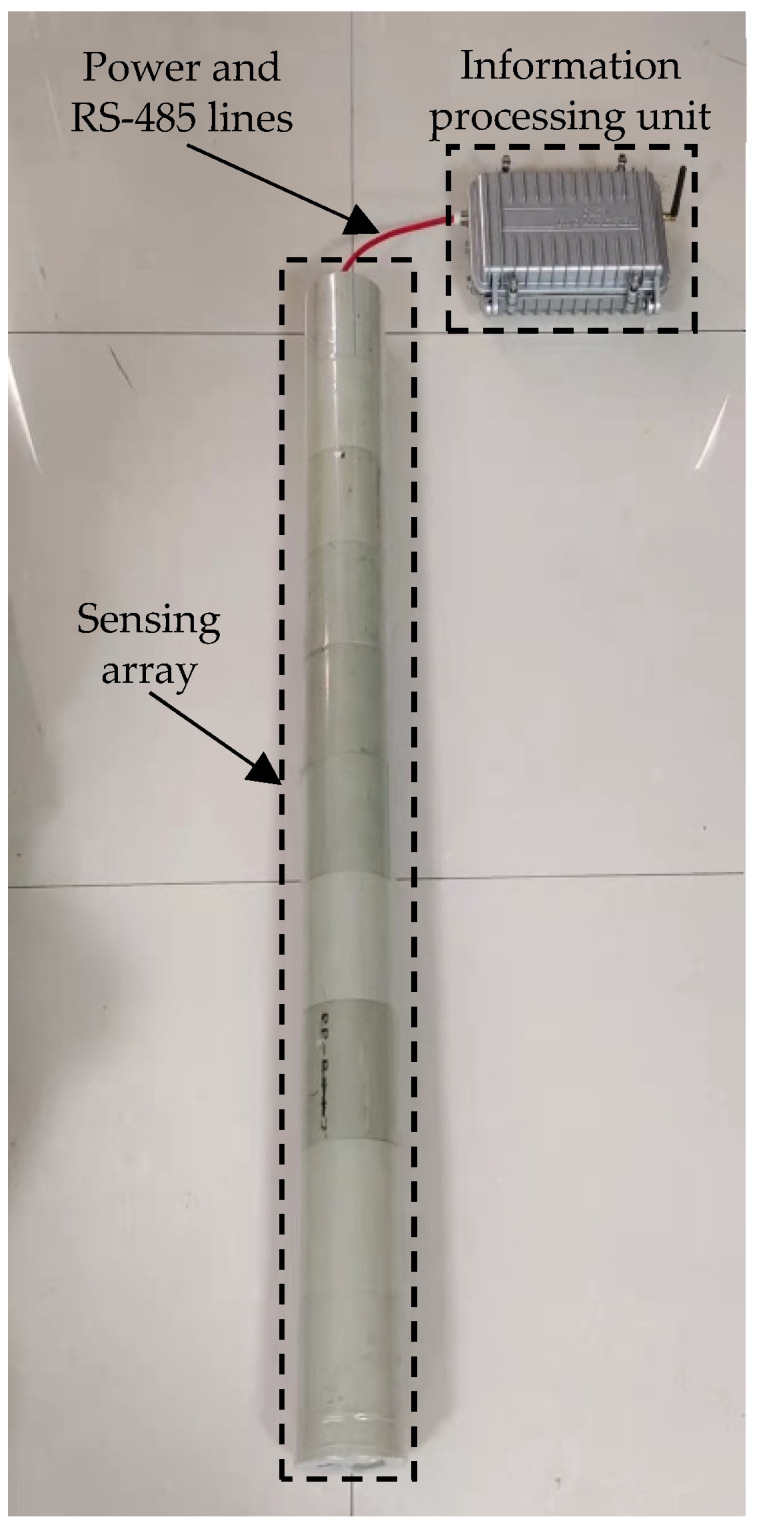
Photo of the underground displacement three-dimensional measurement system.

The three-dimensional measurement system of underground displacement based on the double mutual inductance voltage contour method is mainly composed of a measuring device and remote monitoring platform; between them, data are transmitted through wireless communication technology. As shown in Figure 1, the measuring device mainly consists of an information processing unit and a sensing array connected in series through power lines and RS-485 communication lines.

Equivalent to the host of the measuring device, the information processing unit is responsible for sending measurement signals to the slaves at different addresses, processing the received data through the underground displacement measurement model, and finally sending the measurement data to the Internet of things cloud platform through the 4G communication module.

The sensing array is composed of multiple sensing units. Any of the sensing units is equivalent to a slave of the measuring device and responsible for receiving the measurement signals sent by the information processing unit and performing the corresponding operations. In practical engineering application, the information processing unit works at the surface of the rock and soil mass to be measured, the sensing array is buried vertically into the rock and soil mass through drilling, and the displacement of the landslide is generally related to the displacement of the geodetic coordinate system [43]; therefore the lowest sensing unit in the sensing array is usually buried in the bedrock of the rock and soil mass as the benchmark of the system.

**Figure 2:**
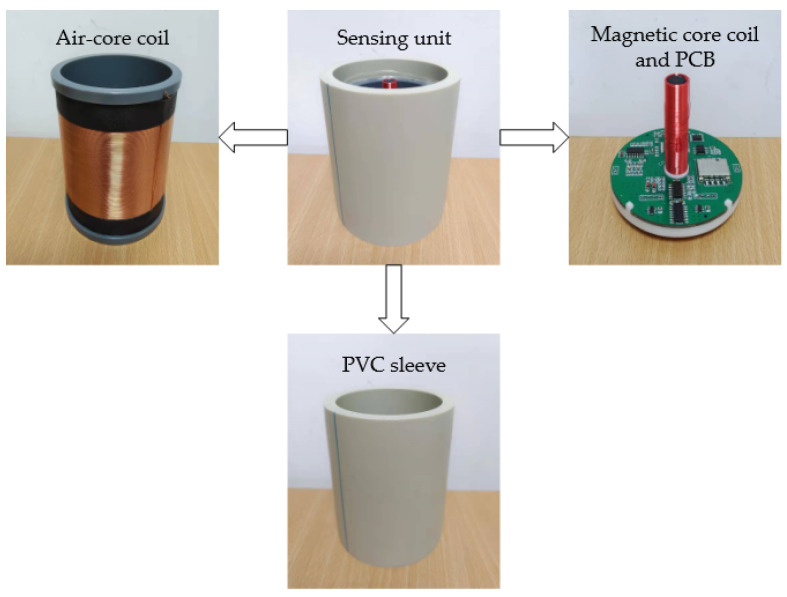
Schematic diagram of the structure of the sensing unit.

Each sensing unit in the array has an identical structure and adopts columnar axisymmetric design. The outside of the sensing unit is a PVC sleeve, which is resistant to deformation and corrosion, and the upper and lower ends of the sensing unit are sealed with glue, which can well protect the internal structure of the sensing unit from impact of underground displacement. The sleeve’s interior comprises the air-core coil, magnetic core coil, and PCB. During installation, it is necessary to ensure that the center points of the air-core coil and the magnetic core coil are on the same plumb lines. The structure of the sensing unit is shown in Figure 2.

### 2.2. Working Principle

**Figure 3:**
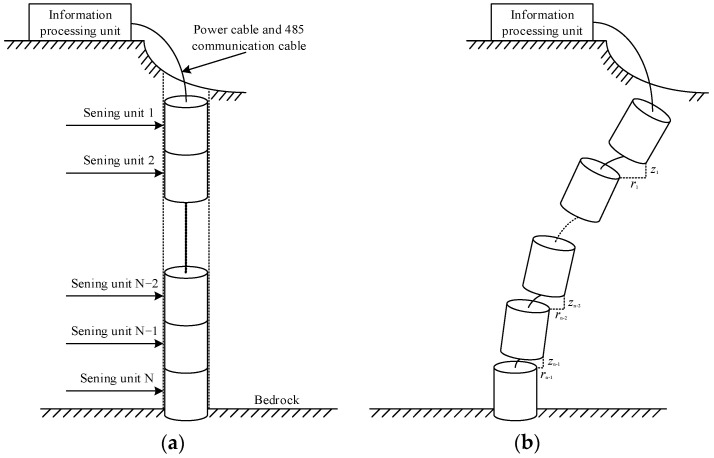
Schematic diagram of the measurement system. (**a**) Non-sliding rock and soil mass; (**b**) Sliding rock and soil mass.

As shown in Figure 3, our proposed underground displacement three-dimensional measuring device takes each measuring unit as a basic unit for measurement. In the sensing array, any two adjacent sensing units form one measuring unit, and N sensing units can constitute N − 1 measuring units. When underground displacement occurs in the rock and soil mass, the rock and soil will drive each sensing unit in the sensing array to produce relative displacement of different sizes and rotation of different angles. Under the control of the information processing unit, the N − 1 measuring units successively measure the relative displacement, tilt angle and azimuth between any two adjacent sensing units from bottom to top. The overall horizontal displacement *r* and vertical displacement *z* of the rock and soil can be estimated by Equation (1), thus realizing the distributed three-dimensional measurement of underground displacement of the rock and soil mass from the deep underground to the surface.

(1) $$\left\{\begin{array}{l} r=\sum_{i=1}^{i=N-1}\Delta r_{i}\\ z=\sum_{i=1}^{i=N-1}\Delta z_{i} \end{array}\right.$$

### 2.3. Measurement Principle

#### 2.3.1. Measurement Principle of Displacement

Our proposed integrated underground displacement sensing unit is designed on electromagnetic mutual inductance. In the previous research, our research team proposed the measurement methods of underground displacement based on single mutual inductance voltage and based on the integration of mutual inductance voltage and Hall voltage, respectively. The former can only achieve displacement measurement in one direction (that is, horizontal displacement measurement), which has certain limitations in practical application. The latter can realize three-dimensional measurement; however, limited by the large temperature drift of Hall sensor; the measurement results are easily affected by environmental factors, which reduces the measurement accuracy. To overcome the limitations of the above underground displacement measurement methods and improve measurement accuracy and stability of the measurement device, a double mutual inductance voltage contour method has been proposed in this paper on virtue of our previous research work.

**Figure 4:**
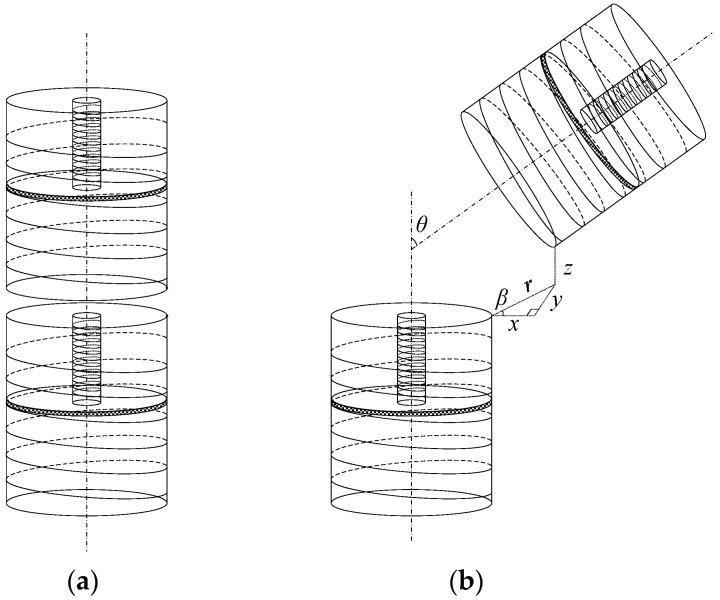
Schematic diagram of the measuring unit based on double mutual inductance voltage contour method. (**a**) Initial state without relative displacement; (**b**) Relative displacement occurred along the azimuth angle *β*.

As an example of one measuring unit in Figure 4, the lower sensing unit is called the excitation end and the upper sensing unit is called the measurement end. When the air-core coil and magnetic core coil in the excitation end are respectively connected with the same sinusoidal signal, two AC signals *u*_I_ and *u*_II_ with the same frequency and different amplitude will be generated on the air-core coil in the measurement end, and *U*_I_ and *U*_II_ will be obtained through the filter rectifier circuit, which is called type I mutual inductance voltage and type II mutual inductance voltage. The calculation equations of *u*_I_ and *u*_II_ are as follows:

(2) $$\left\{\begin{array}{l} u_{I}=\frac{u_{i}}{L_{I}}M_{I}\\ u_{\mathrm{II}}=\frac{u_{i}}{L_{\mathrm{II}}}M_{\mathrm{II}} \end{array}\right.$$

As shown in Equation (2), for each measuring unit, *u*_i_ is the excitation signal successively connected to the air-core coil and the magnetic core coil, *L*_I_ and *L*_II_ are the self-inductance coefficients of air-core coil and magnetic core coil, respectively, *M*_I_ is the mutual-inductance coefficient between the air-core coil at the excitation end and the air-core coil at the measurement end, and *M*_II_ is the mutual-inductance coefficient between the magnetic coil at the excitation end and the air-core coil at the measurement end. In this measurement system, the sensing units in the sensing array exit in the same environment and each sensing unit has exactly the same structure, so the double mutual inductance voltages are only related to the relative position between adjacent sensing units. By applying the principle of electromagnetic mutual inductance, the change of displacement can be converted into the change of mutual-inductance coefficient, and then into the change of mutual inductance voltage; thus the conversion from non-electric quantity to electric quantity is realized. The angle between the Z-axes of two adjacent sensing units, or inter-axial angle *θ*, can be expressed as follows:

(3) $$\theta =\left|\alpha_{A}-\alpha_{B}\right|$$

where *α*_A_ and *α*_B_ are the tilt angles of the excitation end and the measuring end in the measuring unit respectively. By acquiring the data reflecting the double mutual inductance voltages changed with the relative horizontal displacement *r* and vertical displacement *z* between two adjacent sensing units at different inter-axial angle *θ*, the measurement model between the relative displacement to be measured and the measured double mutual inductance voltages for any two adjacent sensing units can be established, so as to characterize the change of the relative position between adjacent sensing units according to the variation of the double mutual inductance voltages.

(4) $$\left\{\begin{array}{l} U_{I}=f_{1}(r,z,\theta )\\ U_{\mathrm{II}}=f_{2}(r,z,\theta ) \end{array}\right.$$

The measurement model at different inter-axial angle *θ* can be applied on the solution of the horizontal displacement *r* and vertical displacement *z* between adjacent sensing units; however, in the actual underground displacement, the moving direction of the sensing unit may be arbitrary and is usually related to the moving direction of rock and soil mass. In order to determine the relative displacement of the sensing units in different directions and realize the three-dimensional measurement of underground displacement, the azimuth *β* is used to indicate the moving direction of rock and soil mass, as shown in Figure 4b. Combined with azimuth *β*, the displacement components of relative horizontal displacement *r* in X-direction and Y-direction can be figured out, thus realizing the distributed three-dimensional measurement of underground displacement of the rock and soil mass from deep underground to the surface. The displacement components in X-direction and Y-direction are shown in the following equations. The measurement principles of azimuth and tilt angle will be presented next.

(5) $$\left\{\begin{array}{l} x=r\cos(\beta )\\ y=r\sin(\beta ) \end{array}\right.$$

#### 2.3.2. Measurement Principle of Azimuth and Tilt Angle

In order to realize the three-dimensional measurement of underground displacement of rock and soil mass, it is necessary not only to obtain the double mutual inductance voltages between adjacent sensing units, but also to determine the tilt angle and azimuth of each sensing unit. The tilt angle can be used to calculate the inter-axis angle between adjacent sensing units to establish the underground displacement measurement model at different inter-axis angles. And the azimuth can be used to judge the moving direction of the sensing unit, thus determining the three-dimensional position change between adjacent sensing units. By solving the above four parameters, we can clearly know the position and state of each sensing unit at a certain moment, so as to realize the three-dimensional distributed measurement of underground displacement of rock and soil mass. The measurement principle of tilt angle *α* and azimuth *β* will be briefed below.

For this measuring device, the attitude variation of any sensing unit in the sensing array can be regarded as a combination of three angles rotating around three rotating axes in sequence in its static state, which are called Euler angles. In order to measure the Euler angles of the sensing unit at any moment, an attitude detecting module including a three-axis magnetometer, a three-axis gyroscope, and a three-axis accelerometer is integrated into each sensing unit, thus capable of measuring the Euler angles *φ*, *ϕ*, and *ψ* along the *X*, *Y*, and *Z* axes in real-time.

**Figure 5:**
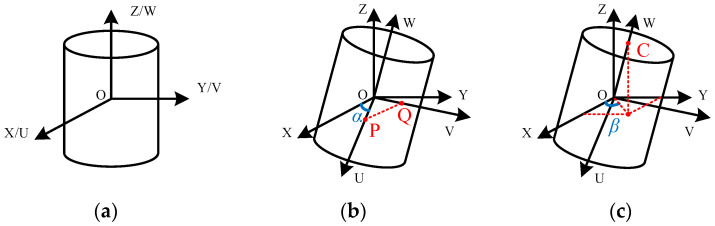
Spatial description of sensing unit. (**a**) Global coordinate system and reference coordinate system; (**b**) Schematics of tilt angle measurement; (**c**) Schematics of azimuth measurement.

As shown in Figure 5a, when a sensing unit is placed horizontally, the global coordinate system O-XYZ and the reference coordinate system O-UVW are established with the internal center point O of the sensing unit as the coordinate origin. The global coordinate system is called coordinate system *A* and the reference coordinate system is called coordinate system *B*. When this sensing unit rotates in any direction, coordinate system *A* will stand still, and coordinate system *B* will change along with the sensing unit. According to the rotation order of the attitude detecting module, the rotated sensing unit can be regarded as a sensing unit in the horizontal state to rotate *ψ* around *Z*-axis first, then rotate *ϕ* around *Y*-axis, and finally rotate *φ* around *X*-axis. These three rotation angles can be directly measured by the attitude detecting module, so the rotation matrix from coordinate system *B* to coordinate system *A* can be solved as follows:

(6) Rz=cos(ψ)−sin(ψ)0sin(ψ)cos(ψ)0001,Ry=cos(ϕ)0sin(ϕ)010−sin(ϕ)0cos(ϕ),Rx=1000cos(φ)−sin(φ)0sin(φ)cos(φ)RBA=Rz⋅Ry⋅Rx=cos(ψ)cos(ϕ)−sin(ψ)cos(φ)+cos(ψ)sin(ϕ)sin(φ)sin(ψ)sin(φ)+cos(ψ)sin(ϕ)cos(φ)sin(ψ)cos(ϕ)cos(ψ)cos(φ)+sin(ψ)sin(ϕ)sin(φ)−cos(ψ)sin(φ)+sin(ψ)sin(ϕ)cos(φ)−sin(ψ)cos(ϕ)sin(φ)cos(ϕ)cos(φ)

Once the rotation matrix is obtained, the tilt angle and azimuth of the sensing unit can be calculated. The measurement principle of the tilt angle is shown in Figure 5b, taking the points P and Q in the *B* coordinate system and finding the coordinates of points P and Q in the *A* coordinate system by multiplying the rotation matrix left, obtaining the coordinates of P and Q points in coordinate system *B* and finding their corresponding coordinates in coordinate system *A* by left-multiplying the rotation matrix as follows:

(7) PA=RBA⋅PB=RBA⋅100=cos(ψ)cos(ϕ)sin(ψ)cos(ϕ)−sin(ψ)

(8) QA=RBA⋅QB=RBA⋅010=−sin(ψ)cos(φ)+cos(ψ)sin(ϕ)sin(φ)cos(ψ)cos(φ)+sin(ψ)sin(ϕ)sin(φ)cos(ϕ)sin(φ)

Therefore, the measurement of the tilt angle of the sensing unit can be converted into the measurement of the inter-angle between the XOY plane and the POQ plane. In the spatial analytic set, the inter-angle between two planes is equivalent to the inter-angle of their respective normal vectors, so the tilt angle *α* of the sensing unit can be solved by Equation (9), where *n*_1_ and *n*_2_ are the normal vectors of XOY plane and POQ plane, respectively.

(9) $$\alpha =\arccos\left(\frac{\vec{n_{1}}\cdot \vec{n_{2}}}{\vec{\left|n_{1}\right|}\cdot \vec{\left|n_{2}\right|}}\right)$$

The measurement principle of azimuth is shown in Figure 5c, obtaining the coordinates of point C in the coordinate system *B* and finding the coordinates of point C in coordinate system *A* by left-multiplying the rotation matrix:

(10) CA=RBA⋅CB=RBA⋅001=−sin(ϕ)−sin(φ)⋅cos(ϕ)cos(φ)⋅cos(ϕ)

The inter-angle between the projection of point C in XOY plane and the X-axis is the azimuth *β*, which can be solved by the following formula:

(11) $$\beta =\arctan\left(\frac{\sin(\varphi )\cdot \cos(\phi )}{\sin(\phi )}\right)$$

## 3. Data Acquisition and Processing

### 3.1. Construction of Experimental Platform

**Figure 6:**
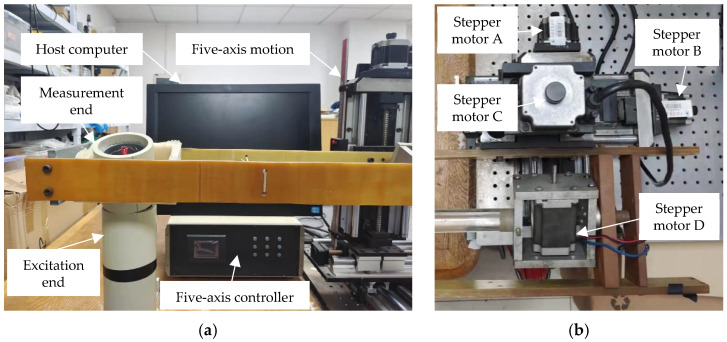
Experimental platform for simulating underground displacement of rock and soil mass. (**a**) System composition; (**b**) Five-axis motion.

It is concluded in the previous sections that the type I and type II mutual inductance voltages are only related to the relative position of any two adjacent sensing units. Therefore, before the sensing array is buried in the rock and soil mass, it is necessary to build an experimental platform to simulate the underground displacement of rock and soil mass to obtain the relationship between the mutual inductance voltages of type I and type II and the relative displacement to be measured. As shown in Figure 6a, the experimental platform is mainly composed of a host computer, a five-axis motion controller, a five-axis motion device, and one measuring unit. The excitation end of the measuring unit is fixed on the smooth horizontal plane, and the measuring end is fixed on the rotating arm of the five-axis motion device. The five-axis motion device is shown in Figure 6b; by controlling the stepping motors A, B, and C, the rotating arm can move towards the X, Y, and Z axes to simulate the three-dimensional change of underground displacement of rock and soil mass, to change the relative position and relative moving directions between adjacent sensing units. By controlling the stepper motor D, the rotation degree and direction of the rotating arm can be changed, and then the tilt angle of the measuring end in the measuring unit can be changed so that data acquisition experiments at different inter-axis angles can be performed. Since the structure of each sensing unit in the sensing array is identical and is designed in the axisymmetric structure, it is reasonable to perform data acquisition experiments only on one measuring unit in the sensing array. To simplify the experiment process, the azimuth of the sensing units is set to zero, *β* = 0°, which means *x* = *r*cos *β* = *r*, *y* = *r*sin *β* = 0.

In the data acquisition experiment, the initial position between adjacent sensing units is shown in Figure 6a, where both the relative horizontal displacement and vertical displacement between adjacent sensing units are 0 mm, and the automatic measurement is realized under the control of the host computer. During the measurement process, the host computer will send measurement instructions to the excitation end and the measurement end, in turn, to collect type I and II mutual inductance voltages and change the relative displacement between adjacent sensing units and the inter-axis angle through the five-axis motion device, to collect the double mutual inductance voltages at different inter-axis angles and positions, which are called the double mutual inductance voltage datasets. The measurement range of horizontal displacement and vertical displacement is 0–50 mm, and the change step is 1 mm. The variation range of inter-axis angle is 0°–80°, and the variation interval is 5°. Since the excitation end is fixed on the horizontal plane, the inter-axis angle between adjacent sensing units is equal to the tilt angle of the measurement end.

### 3.2. Double Mutual Inductance Voltage Contour

**Figure 7:**
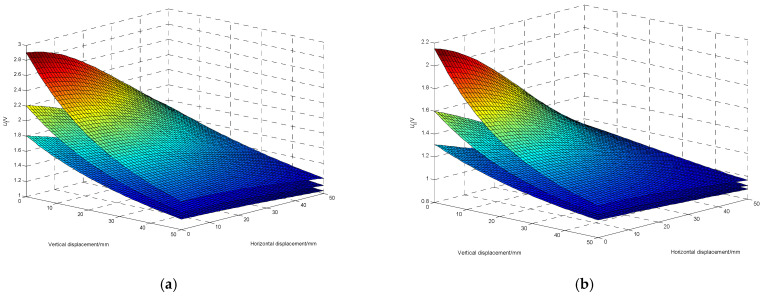
Three-dimensional graph of (**a**) type I mutual inductance voltage, (**b**) type II mutual inductance voltage versus horizontal displacement and vertical displacement at different inter-axis angles. (10°, 30°, and 50° from top to bottom).

The data acquisition experiment can obtain the double mutual inductance voltage datasets at different inter-axis angles. Figure 7a,b are three-dimensional graphs depicting the relationship among the relative horizontal displacement, relative vertical displacement, and the mutual inductance voltage of type I (*U*_I_) and type II (*U*_II_) respectively at different inter-axis angles, which are 10°, 30° and 50° from top to bottom.

**Figure 8:**
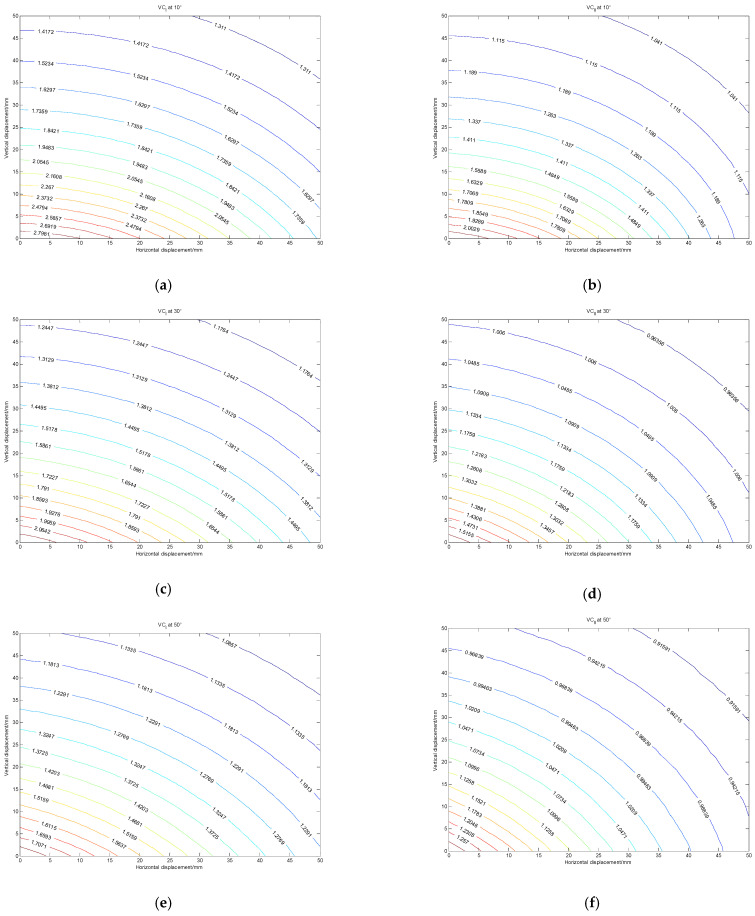
Double mutual inductance voltage contours at different inter-axis angles. (**a**) VC_I_ at 10°; (**b**) VC_II_ at 10°; (**c**) VC_I_ at 30°; (**d**) VC_II_ at 30°; (**e**) VC_I_ at 50°; (**f**) VC_II_ at 50°.

From Figure 7, it can be seen that type I and type II mutual inductance voltages are not equal under the same position, and both type I and type II mutual inductance voltages will decrease with the increase of the inter-axis angle. At the same inter-axis angle, both type I and type II mutual inductance voltages will decrease with the increase of relative displacement between adjacent sensing units, and the double mutual inductance voltage measured at a certain position will correspond to multiple different data pairs of horizontal displacement and vertical displacement, [*r*, *z*]. The corresponding coordinate points of these data pairs are called equivalent discrete points of mutual inductance voltage, the points of equal mutual inductance voltage are called the mutual inductance voltage equivalence discrete points, and the curve composed of multiple equivalent discrete points of mutual inductance voltages is called the mutual inductance voltage contour. By projecting the three-dimensional surface of the double mutual inductance voltages onto the two-dimensional plane, the double mutual inductance voltage contours at different inter-axis angles can be obtained. Figure 8 shows type I and type II mutual inductance voltage contours (abbreviated as VC_I_ and VC_II_ respectively) at the inter-axis angles of 10°, 30°, and 50°, in which the horizontal axis represents the horizontal displacement and the vertical axis represents the vertical displacement.

It can be found from Figure 8 that the value of type I or type II mutual inductance voltage measured at a certain position will correspond to multiple different positions in the contour. The relative displacement between adjacent sensing units at the current time cannot be accurately judged only by one voltage contour of type I or type II. Therefore, the double mutual inductance voltage contour method is proposed in this paper. By virtue of the different self-inductance coefficients of the air-core coil and magnetic core coil, two mutual inductance voltage contours with different variation trends can be obtained from the mutual inductance voltages of type I and II at a particular position. These two curves have and only have one intersection, and the intersection coordinates correspond to the relative displacement between these two adjacent sensing units. Through this method, the relative displacement between adjacent sensing units can be converted into the intersection coordinates of type I and type II voltage contours, thus realizing the conversion from displacement to voltage.

## 4. Establishment of Underground Displacement Three-Dimensional Measurement Model

The establishment of a three-dimensional measurement model of underground displacement based on the double mutual inductance voltage contour method mainly includes three steps:

1. The data acquisition experiment is carried out on the experimental platform to obtain the double mutual inductance voltage datasets at different inter-axis angles and the double mutual inductance voltages, inter-axis angle and azimuth in the measuring unit are measured under the control of the information processing unit.
2. Applying the interpolation theory, the dataset of double mutual inductance voltages at the current inter-axis angle between two adjacent sensing units at the current moment is obtained, and the equivalent discrete points of double mutual inductance voltages are solved according to the values of double mutual inductance voltages.
3. Applying the equivalent discrete points of double mutual inductance voltages to construct two contours of double mutual inductance voltages. These two contours have only one intersection. The intersection coordinates, corresponding to the horizontal displacement and vertical displacement between these two adjacent sensing units, can be solved by numerical analysis method.

The steps for building the measurement model are described in detail in the following subsections.

### 4.1. Establishment of Double Mutual Inductance Voltage Datasets

The underground displacement three-dimensional measurement model is established based on the double mutual inductance voltage datasets under different inter-axis angles, so the variation of inter-axis angles between adjacent sensing units is one of the important factors that change the measurement results. In the actual underground displacement, the variation of the inter-axis angles between adjacent sensing units is arbitrary and usually is accompanied by the change of underground displacement of rock and soil mass, so the double mutual inductance voltage datasets corresponding to each measuring unit will change accordingly. The purpose of improving the measurement accuracy of the system can be realized by collecting the double mutual inductance voltage datasets at any inter-axis angles and storing them in the microprocessor of the information processing unit. However, this will not only greatly increase the workload of data acquisition experiments, but also lead to work difficulties: limited by the memory capacity of the microprocessor, only a limited number of datasets can be stored. To broaden the measurement range of the underground displacement three-dimensional measurement sensor, it is required that the variation ranges of the relative displacement and inter-axis angle between adjacent sensing units are quite large, so it is virtually impossible to use a definite interpolation polynomial to depict the variation relationship between the double mutual inductance voltages and the inter-axis angle at the same position for the measuring unit. After comparison, the linear interpolation method is adopted. This method has a small amount of calculation. It only needs to carry out several groups of data acquisition experiments on each measuring unit under different inter-axis angles to build the datasets under any inter-axis angle with quite high accuracy. The main steps are as follows:

1. Data acquisition experiment for measuring units at different inter-axis angles is carried out to obtain the corresponded datasets of double mutual inductance voltages. Since increasing the number of datasets can also improve the accuracy of the linear interpolation method, so on the premise of not exceeding the memory size of the information processing unit, the variation range of the inter-axis angle in the data acquisition experiment sets as 0° to 80° and the variation spacing sets as 5°.
2. Under the control of the information processing unit, the tilt angles of both the excitation end and the measurement end in each measuring unit are first solved through the attitude angle solving formula, and then converted into the inter-axis angle for this measuring unit. Find two datasets adjacent to this inter-axis angle, then calculate the double mutual inductance voltage datasets under the current inter-axis angle through the following formula:

(12) $$\left\{\begin{array}{l} U_{I\mathrm{ij}(\theta )}=U_{I\mathrm{ij}(\gamma )}-\frac{\left(U_{I\mathrm{ij}(\gamma )}-U_{I\mathrm{ij}(\eta )}\right)}{5}\cdot (\theta -\gamma )\\ U_{\mathrm{II}\mathrm{ij}(\theta )}=U_{\mathrm{II}\mathrm{ij}(\gamma )}-\frac{\left(U_{\mathrm{II}\mathrm{ij}(\gamma )}-U_{\mathrm{II}\mathrm{ij}(\eta )}\right)}{5}\cdot (\theta -\gamma ) \end{array}\right.$$

where *θ* is the current inter-axis angle between these two adjacent sensing units, *γ* and *η* are the inter-axis angles of these two datasets adjacent to *θ*, satisfying *η* − *γ* = 5°.

### 4.2. Calculation of Double Mutual Inductance Voltage Equivalent Discrete Points

**Figure 9:**
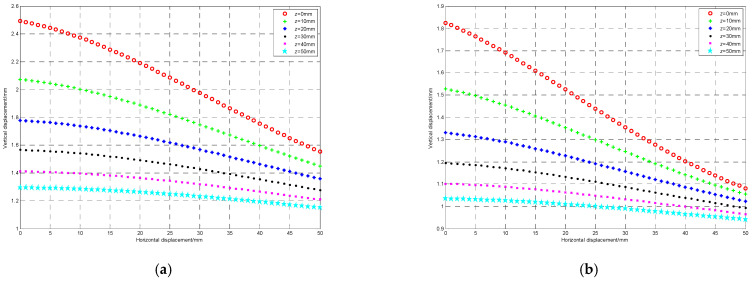
The relationship curve between the horizontal displacement and the double mutual inductance voltages. (**a**) Mutual inductance voltage of Type I; (**b**) mutual inductance voltage of Type II.

The double mutual inductance voltage contour is composed of multiple equivalent voltage points. When the vertical displacement between adjacent sensing units remains unchanged, the relationship between the horizontal displacement and the mutual inductance voltage of type I and type II respectively at different inter-axis angles is shown in Figure 9, where the vertical displacement corresponding to the six curves is 0 mm, 10 mm, 20 mm, 30 mm, 40 mm and 50 mm from top to bottom.

It can be observed from Figure 9 that when the vertical displacement is constant, both type I and type II mutual inductance voltages will decrease with the increase of horizontal displacement, and the adjacent two points can be approximately regarded as linear variation. Therefore, the piece-wise linear method is proposed to solve the equivalent discrete points of double mutual inductance voltages, that is, first, the horizontal displacement under the same vertical displacement is divided into several different intervals, [*r*_ij_, *r*_(i+1)j_], (i = 0, 1, …, m − 1), (j = 0, 1, …, n), m and n are the measurement ranges of horizontal displacement and vertical displacement respectively, and then the contours of mutual inductance voltage are approximately replaced by straight lines in each interval. Taking type I mutual inductance voltage as an example, when *U*_Iij_ < *U*_I_ < *U*_I(i+1)j_, the horizontal displacement corresponding to the voltage under any vertical displacement can be obtained through Equation (13).

(13) $$r=\frac{U_{I\mathrm{ij}}-U_{I}}{U_{I\mathrm{ij}}-U_{I(i+1)\;j}}+i,\;\left(U_{I}\in \left[U_{I\mathrm{ij}},U_{I(i+1)j}\right]\right)$$

where i and j represent the variation intervals of horizontal displacement *r* and vertical displacement *z*, respectively. By changing the vertical displacement successively, it is possible to find multiple equivalent voltage discrete points corresponding to this voltage within the whole measurement range, laying a foundation for the next curve solution.

The double mutual inductance voltage equivalent discrete points of two adjacent sensing units at a certain position can be obtained by the mentioned linear interpolation method and piece-wise linear method. For example, when the inter-axis angle between two adjacent sensing units is 20°, and both the relative horizontal displacement and vertical displacement are 15 mm, then the measured mutual inductance voltages of type I and type II are 1.819 V and 1.328 V, respectively. The equivalent discrete points of mutual inductance voltages of type I and type II obtained by the piece-wise linear method are shown in Table 1.

### 4.3. Displacement Solving

**Figure 10:**
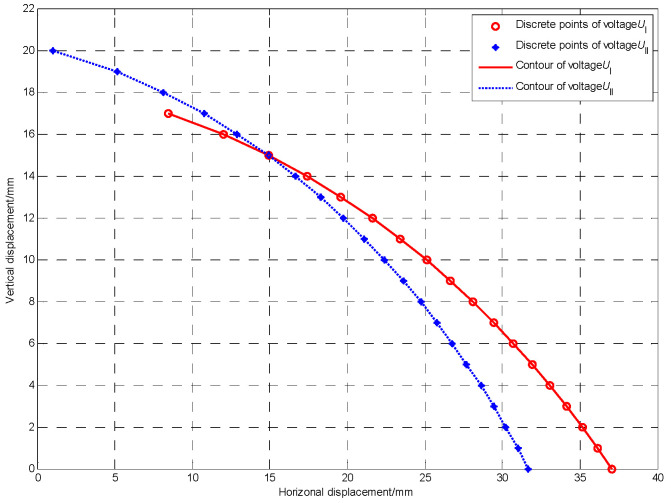
Two contours correspond to the double mutual inductance voltages of 1.819 V (*U*_I_) and 1.328 V (*U*_II_) when the inter-axis angle is 20°.

**Figure 11:**
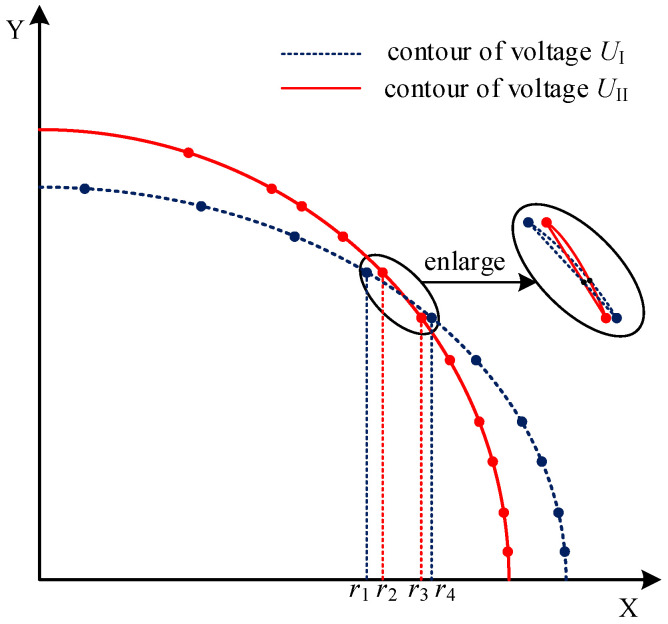
Contour and intersection interval of type I and type II mutual inductance voltages.

The equivalent voltage discrete points in Table 1 are connected with smooth curves to form the double mutual inductance voltage contours shown in Figure 10. It can be observed that these two contours have only one intersection, so the case of Figure 11 will exist. There is a certain interval [*r*_1_, *r*_4_] and [*r*_2_, *r*_3_] within the intersection range near the double mutual inductance voltage contours, and can be called the intersection interval. The former interval represents the horizontal displacement of mutual inductance voltage contour of type I, and the later interval represents that of type II. The intersection of the mutual inductance voltage contours of type I and type II lies in this interval.

In order to solve the intersecting coordinates of the double mutual inductance voltage contours, namely, the relative displacement between adjacent sensing units, the curve expressions of type I and type II mutual inductance voltage contours need to be obtained according to the discrete points of double mutual inductance voltage equivalence. The commonly used methods are the interpolation method and the curve-fitting method. Lagrange interpolation is a standard polynomial interpolation method and can calculate the curve expression passing through all discrete points according to the known discrete points. The Lagrange interpolation polynomial is as follows:

(14) $$L_{n}(x)=y_{0}l_{0}(x)+y_{1}l_{1}(x)+\cdots +y_{n}l_{n}(x)=\sum_{i=0}^{n}y_{i}l_{i}(x)$$

where *l*_i_(*x*) is the interpolation basis function with the following calculation expression:

(15) $$l_{i}(x)=\frac{\left(x-x_{0})\cdots (x-x_{i-1})(x-x_{i+1})\cdots (x-x_{n}\right)}{\left(x_{i}-x_{0})\cdots (x_{i}-x_{i-1})(x_{i}-x_{i+1})\cdots (x_{i}-x_{n}\right)},\;i=0,1,\cdots ,n$$

*x*_i_ is the abscissa of the discrete interpolation point, and *y*_i_ is the ordinate of the discrete interpolation point.

In the case of Figure 11, taking three equivalent discrete points in the intersection interval as interpolation points, the contour equations of type I and type II mutual inductance voltages obtained by the Lagrange interpolation method are as follows:

(16) $$\left\{\begin{array}{l} z_{I}=-0.011637r^{2}-0.043357r+18.1279\\ z_{\mathrm{II}}=-0.0210226r^{2}+0.086933r+18.3719 \end{array}\right.$$

According to the double mutual inductance voltage contour equations expressed in Equation (16), the intersection coordinates can be solved. As mentioned earlier, the solved intersection coordinates correspond to the horizontal displacement *r* and vertical displacement *z* between these two adjacent sensing units:

*r* = 14.879 mm, *z* = 15.011 mm

The least-squares fitting method is a standard curve fitting method, in which the fitting formula can be expressed as follows:

(17) $$S(x)=a_{0}\varphi_{0}(x)+a_{1}\varphi_{1}(x)+\cdots +a_{n}\varphi_{n}(x)=\sum_{i=0}^{n}a_{i}\varphi_{i}(x)$$

where *φ*_i_(*x*) is a selected set of linearly independent functions and *a*_i_ is the undetermined coefficient (i = 0, 1, …, n) and n is less than the number of discrete point sets.

Select the same intersection interval as shown in Figure 11, then the contour equations of type I and type II mutual inductance voltages obtained by the least-squares fitting method are as follows:

(18) $$\left\{\begin{array}{l} z_{I}=-0.02102r^{2}+0.08693r+18.37\\ z_{\mathrm{II}}=-0.01116r^{2}-0.04336r+18.13 \end{array}\right.$$

Similarly, the intersection coordinates [*r*, *z*] can be solved according to the double mutual inductance voltage contour equations expressed in Equation (18).

*r* = 14.853 mm, *z* = 15.024 mm

Our research shows that both the Lagrange interpolation method and least-squares curve fitting method can solve the curve equation according to the discrete points to determine the relative displacement between adjacent sensing units; however, both methods need a lot of calculation, and multiple solutions will be introduced when solving the high-order equations. Therefore, an interval-linear method is proposed in this paper; as shown in Figure 11, the curve segment in the intersection interval is approximately regarded as linear. In this way, the expressions of two linear equations can be derived from the coordinates of the endpoints of the intersection interval. The intersection coordinates of the two linear equations are taken as the intersection coordinates of the contours of double mutual inductance voltages, which correspond to the horizontal and vertical displacements between the adjacent sensing units.

To fully evaluate the validity and accuracy of these methods, the Lagrange interpolation method, the least-squares fitting method, and the interval-linear method are used for experimental verification in the intersection interval where the inter-axis angle is 20°, and the measurement results are shown in Table 2. Statistical analysis of the measurement results shows: (1) Applying the Lagrange interpolation method, the max error, relative average error, and variance of [*r*, *z*] is [−0.500 mm, +0.499 mm], [−0.168 mm, +0.112 mm], [0.048 mm, 0.048 mm], respectively. Applying the least-squares fitting method, they are [−0.435 mm, +0.448 mm], [−0.166 mm, +0.111 mm], [0.043 mm, 0.044 mm], respectively. And applying the interval-linear method, they are [−0.439 mm, +0.439 mm], [−0.097 mm, +0.042 mm], [0.018 mm, 0.025 mm], respectively. (2) The max error, relative average error, or the variance of the measurement results obtained by the interval linear method are smaller than the other two methods. It indicates that the interval linear method has higher measurement accuracy and less calculation, so we finally adopt this method to solve the intersection coordinates of the double mutual inductance voltage contours.

## 5. Results and Discussion

To evaluate our proposed underground displacement three-dimensional measurement model, the validation experiments are conducted on the experimental platform described in Section 3.1. Several inter-axis angles (18.35°, 36.34°, 62.82°) are arbitrarily selected under the consistent experimental conditions, and the comparisons between the measured values and the experimental testing values (actual values) are shown in Table 3. From the table, it can be seen that the maximum errors of horizontal displacement and vertical displacement solved by this measurement model will not exceed 1 mm, and most of the errors are within 0.5 mm, thus verifying the measurement accuracy and reliability for our proposed underground displacement three-dimensional measuring model, and the rationality and efficiency for our proposed underground displacement three-dimensional measurement method.

Anti-interference ability is an important factor in measuring whether a device is reliable, and it is essential to eliminate or reduce the noise effect on the measurement system. The noise source of the device is related to its environment. Our proposed underground displacement monitoring sensor can be divided into data acquisition stage and engineering application stage according to the different application environments.

In the data acquisition stage, the random errors caused by external factors mainly include two types: one is caused by the stepper motor in the experimental platform, and the other is caused by the temperature drift of the components in the hardware circuit. We take the following measures to effectively reduce the impact of noise on the device. Firstly, we choose the stepper motor with high displacement resolution, and the maximum movement error will not exceed 0.01 mm, which can be basically ignored. Secondly, we selected high-precision and low-temperature drift chips and components in the hardware circuit [44]. We further eliminated the random error caused by temperature by sampling the mutual inductance voltage for many times and taking the average value. These measures ensure that the noise will not greatly impact the measurement system in the data acquisition stage and ensure the reliability of the double mutual inductance voltage datasets.

In the engineering application stage, in order to ensure that the measurement system can work stably, we need to consider the influence of rock and soil geological environment on the sensing unit. The factors that trigger landslides mainly include soil porosity, soil depth, soil water content, and landslide velocity [45,46,47]. Soil water content requires good water-resistance of the sensing unit, soil depth and soil porosity require good pressure resistance of the sensing unit, and landslide velocity requires high displacement sensitivity and fast solution speed of the sensing unit. Firstly, the measurement device adopts a flexible structure and the principle of electromagnetic mutual inductance so that the sensing unit has high displacement sensitivity. Secondly, the mathematical manipulations of the underground displacement measurement model are completed by the high-speed MCU in each sensing unit, which makes the sensing unit have a fast solution speed. Finally, the outer wall of the sensing unit is made of PVC sleeve that is not easily deformed, and the upper and lower ends of the sensing unit are sealed with glue so that the sensing unit has very good waterproof and pressure resistance performance. Therefore, the above factors will not greatly impact the measurement system.

## 6. Conclusions

Landslide is widely distributed and frequently occur all over the world. It is one of the most severe geological disasters affecting human activities and the safety of life and property. To grasp the three-dimensional deformation characteristics and evolution dynamics of landslide rock and soil mass from the surface to the deep underground in real-time to predict the disaster trend and scope more timely and accurately, and to overcome the limitations of the existing underground displacement monitoring technology, a new underground displacement three-dimensional measurement method based on the principle of intersection-solving of double mutual inductance voltage contours is proposed in this paper. Compared to the currently available underground displacement measurement technology, our presented method has the following characteristics:

1. Flexible structure design. One characteristic of our proposed underground displacement three-dimensional measurement device is that it has a flexible sensing array structure composed of multiple sensing units in series. Compared with the rigid structure of inclinometer pipe [21], the flexible structure has better coupling with landslide displacement, higher displacement sensitivity. It can reflect the landslide change in real-time to play a better early warning and prediction function.
2. Automatic measurement. When the system works, under the control of the information processing unit, it can measure the horizontal displacement, vertical displacement, inter-axis angle, and azimuth between any two adjacent sensing units from bottom to top to realize the distributed three-dimensional measurement of underground displacement of rock and soil mass from deep underground to surface.
3. Three-dimensional measurement. Borehole inclinometer [21,22], TDR [23,24], and fiber optic sensing technology [25] cannot achieve three-dimensional measurement of underground displacement. The system adopts the principle of electromagnetic mutual inductance to realize three-dimensional measurement. It has stronger stability and a wider effective measurement range compared with the measurement principle of the above underground displacement monitoring technology. Through theoretical analysis and experimental verification, it is found that the horizontal displacement and vertical displacement between adjacent sensing units cannot be accurately determined by only one mutual inductance voltage. Therefore, the double mutual inductance contour method is proposed in this paper. Each sensing unit includes an air-core, a magnetic core coil, and an attitude detecting module. The position information of adjacent sensing units can be characterized by the double mutual inductance voltages and inter-axis angles.
4. High measurement accuracy. Several data acquisition experiments of type I and type II mutual inductance voltages under different inter-axis angles between adjacent sensing units are conducted, where the relationship between the measured mutual inductance voltages of type I and type II. The relative measuring displacement between any two adjacent sensing units under different inter-axis angles is investigated. A new three-dimensional measurement model of underground displacement is proposed based on the principle of interval-interpolation and contour-modeling. Through this method, the double mutual inductance voltage and the Euler angle of three axes measured at any time and position can be transformed into the horizontal displacement, vertical displacement, inter-axis angle and azimuth between any two adjacent sensing units. To fully evaluate the effectiveness and accuracy of the measurement model, a series of comparative experiments between the measured displacement and the actual displacement have been conducted on the self-designed underground displacement three-dimensional measurement experimental device. The experimental results show that the maximum errors of horizontal displacement and vertical displacement based on the proposed measurement model are less than 1 mm in the inter-axis angle range from 0° to 80°, which has higher measurement accuracy and reliability compared with other underground displacement monitoring technologies [4,20] and meets the accuracy and reliability requirements of landslide displacement monitoring.

## Figures and Tables

**Table 1:** Equivalent discrete point of double mutual inductance voltages when the relative displacement is [15, 15] at 20° inter-axis angle (unit: mm).

Equivalent Discrete Point of Type I Mutual Inductance Voltage	Equivalent Discrete Point of Type II Mutual Inductance Voltage
[37.074, 0.000] [36.160, 1.000] [35.160, 2.000] [34.153, 3.000] [33.087, 4.000] [31.909, 5.000] [30.714, 6.000] [29.429, 7.000] [28.105, 8.000] [26.650, 9.000] [25.111, 10.000] [23.412, 11.000] [21.600, 12.000] [19.571, 13.000] [17.385, 14.000] [14.909, 15.000] [12.000, 16.000] [8.428, 17.000]	[31.684, 0.000] [31.000, 1.000] [30.211, 2.000] [29.444 3.000] [28.611, 4.000] [27.667, 5.000] [26.765, 6.000] [25.800, 7.000] [24.733, 8.000] [23.642, 9.000] [22.385, 10.000] [21.083, 11.000] [19.750, 12.000] [18.250, 13.000] [16.636, 14.000] [14.900, 15.000] [12.889, 16.000] [10.750, 17.000] [8.143, 18.000] [5.167, 19.000] [1.000, 20.000]

**Table 2:** Comparison of actual displacement [*r*, *z*] and measured displacement [*r*, *z*] under different methods when the inter-axis angle is 20°. (*r*-horizontal displacement in mm, *z*-vertical displacement in mm).

Actual Displacement	Measured Displacement		
	Lagrange Interpolation	Least-Squares Fitting	Interval Linear
[5, 5]	[4.882, 4.983]	[4.883, 4.982]	[4.902, 4.969]
[10, 10]	[9.964, 9.936]	[9.961, 9.935]	[9.976, 9.919]
[15, 15]	[14.879, 15.011]	[14.853, 15.024]	[14.923, 14.915]
[20, 20]	[19.817, 20.036]	[19.811, 20.041]	[19.837, 20.029]
[25, 25]	[25.023, 24.887]	[25.044, 24.877]	[25.019, 24.883]
[30, 30]	[30.221, 29.935]	[30.203, 29.947]	[30.034, 30.026]
[35, 35]	[34.640, 35.300]	[34.619, 35.320]	[34.919, 35.067]
[40, 40]	[39.558, 40.424]	[39.565, 40.421]	[39.957, 40.130]
[45, 45]	[44.500, 45.499]	[44.570, 45.448]	[44.561, 45.439]
Max Error	[−0.500, +0.499]	[−0.435, +0.448]	[−0.439, +0.439]
Relative Average Error	[−0.168, +0.112]	[−0.166, +0.111]	[−0.097, +0.042]
Variance	[0.048, 0.048]	[0.043, 0.044]	[0.018, 0.025]

**Table 3:** Comparison of actual displacement [*r*, *z*] and measured displacement [*r*, *z*] when the inter-axis angle is 18.35°, 36.34°, and 62.82°, respectively (unit: mm).

Actual Displacement	Measured Displacement		
	*θ* = 18.35°	*θ* = 36.34°	*θ* = 62.82°
[5, 5]	[5.07, 4.47]	[5.01, 4.87]	[5.07, 5.18]
[10, 10]	[9.93, 9.66]	[9.85, 9.92]	[10.30, 10.11]
[15, 15]	[14.85, 14.71]	[14.90, 14.96]	[15.33, 15.31]
[20, 20]	[19.85, 19.70]	[19.99, 20.01]	[20.30, 20.27]
[25, 25]	[24.79, 24.72]	[25.04, 25.06]	[25.30, 25.25]
[30, 30]	[29.72, 29.70]	[30.01, 29.74]	[30.51, 30.44]
[35, 35]	[34.85, 34.75]	[34.91, 34.88]	[35.20, 35.26]
[40, 40]	[39.97, 39.87]	[40.28, 39.79]	[40.60, 40.02]
[45, 45]	[44.65, 44.90]	[45.31, 44.78]	[45.59, 45.44]
Max Error	[−0.35, −0.53]	[+0.31, −0.26]	[+0.60, +0.44]
Relative Average Error	[−0.15, −0.28]	[+0.03, −0.11]	[+0.36, +0.25]
Variance	[0.014, 0.014]	[0.023, 0.01]	[0.028, 0.017]

## References

[1] Wu Y., Niu R., Wang Y., Chen T. (2020). A Fast Deploying Monitoring and Real-Time Early Warning System for the Baige Landslide in Tibet, China. Sensors.

[2] Biagi L., Grec F.C., Negretti M. (2016). Low-Cost GNSS Receivers for Local Monitoring: Experimental Simulation, and Analysis of Displacements. Sensors.

[3] Ma J., Liu X., Niu X., Wang Y., Wen T., Zhang J., Zou Z. (2020). Forecasting of Landslide Displacement Using a Probability-Scheme Combination Ensemble Prediction Technique. Int. J. Environ. Res. Public Health.

[4] Wang K., Zhang S., Chen J., Teng P., Wei F., Chen Q. (2017). A Laboratory Experimental Study: An FBG-PVC Tube Integrated Device for Monitoring the Slip Surface of Landslides. Sensors.

[5] Wang Y., Tang H., Wen T., Ma J. (2020). Direct Interval Prediction of Landslide Displacements Using Least Squares Support Vector Machines. Complexity.

[6] Ma J., Niu X., Tang H., Wang Y., Wen T., Zhang J. (2020). Displacement Prediction of a Complex Landslide in the Three Gorges Reservoir Area (China) Using a Hybrid Computational Intelligence Approach. Complexity.

[7] Ma J.W., Tang H.M., Liu X., Wen T., Zhang J.R., Tan Q.W., Fan Z.Q. (2017). Probabilistic forecasting of landslide displacement accounting for epistemic uncertainty: A case study in the Three Gorges Reservoir area, China. Landslides.

[8] Zhang Q., Wang Y., Sun Y., Gao L., Zhang Z., Zhang W., Zhao P., Yue Y. (2016). Using Custom Fiber Bragg Grating-Based Sensors to Monitor Artificial Landslides. Sensors.

[9] Squarzoni C., Delacourt C., Allemand P. (2005). Differential single-frequency GPS monitoring of the La Valette landslide (French Alps). Eng. Geol..

[10] Schlögel R., Doubre C., Malet J.-P., Masson F. (2015). Landslide deformation monitoring with ALOS/PALSAR imagery: A D-InSAR geomorphological interpretation method. Geomorphology.

[11] Jaboyedoff M., Oppikofer T., Abellán A., Derron M.-H., Loye A., Metzger R., Pedrazzini A. (2012). Use of LIDAR in landslide investigations: A review. Nat. Hazards.

[12] Corsini A., Bonacini F., Mulas M., Petitta M., Ronchetti F., Truffelli G. (2015). Long-Term Continuous Monitoring of a Deep-Seated Compound Rock Slide in the Northern Apennines (Italy). Engineering Geology for Society and Territory.

[13] Gili J.A., Corominas J., Rius J. (2000). Using Global Positioning System techniques in landslide monitoring. Eng. Geol..

[14] Manconi A. (2012). Surface displacements following the Mw 6.3 L’Aquila earthquake: One year of continuous monitoring via Robotized Total Station. Ital. J. Geosci..

[15] Karimzadeh S., Matsuoka M. (2020). Ground Displacement in East Azerbaijan Province, Iran, Revealed by L-band and C-band InSAR Analyses. Sensors.

[16] Notti D., Cina A., Manzino A., Colombo A., Bendea I.H., Mollo P., Giordan D. (2020). Low-Cost GNSS Solution for Continuous Monitoring of Slope Instabilities Applied to Madonna Del Sasso Sanctuary (NW Italy). Sensors.

[17] Caviedes-Voullième D., Juez C., Murillo J., García-Navarro P. (2014). 2D dry granular free-surface flow over complex topography with obstacles. Part I: Experimental study using a consumer-grade RGB-D sensor. Comput. Geosci..

[18] Juez C., Caviedes-Voullième D., Murillo J., García-Navarro P. (2014). 2D dry granular free-surface transient flow over complex topography with obstacles. Part II: Numerical predictions of fluid structures and benchmarking. Comput. Geosci..

[19] Nichols A., Rubinato M. (2016). Low-cost 3D mapping of turbulent flow surfaces. Sustainable Hydraulics in the Era of Global Change.

[20] Zhang Y., Tang H., Lu G., Wang Y., Li C., Zhang J., An P., Shen P. (2020). Design and Testing of Inertial System for Landslide Displacement Distribution Measurement. Sensors.

[21] Simeoni L., Mongiovì L. (2007). Inclinometer monitoring of the Castelrotto landslide in Italy. J. Geotech. Geoenviron. Eng..

[22] Zhang Y., Tang H., Li C., Lu G., Cai Y., Zhang J., Tan F. (2018). Design and Testing of a Flexible Inclinometer Probe for Model Tests of Landslide Deep Displacement Measurement. Sensors.

[23] Stangl R., Buchan G., Loiskandl W. (2009). Field use and calibration of a TDR-based probe for monitoring water content in a high-clay landslide soil in Austria. Geoderma.

[24] Su M.-B., Chen I.-H., Liao C.-H. (2009). Using TDR cables and GPS for landslide monitoring in high mountain area. J. Geotech. Geoenviron. Eng..

[25] Zhu H.H., Shi B., Zhang C.C. (2017). FBG-Based Monitoring of Geohazards: Current Status and Trends. Sensors.

[26] Fosalau C., Zet C., Petrisor D. (2015). Multiaxis Inclinometer for in Depth Measurement of Landslide Movements. Sens. Rev..

[27] Ruzza G., Guerriero L., Revellino P., Guadagno F.M. (2020). A Multi-Module Fixed Inclinometer for Continuous Monitoring of Landslides: Design, Development, and Laboratory Testing. Sensors.

[28] Chung C.-C., Lin C.-P. (2019). A comprehensive framework of TDR landslide monitoring and early warning substantiated by field examples. Eng. Geol..

[29] Picarelli L., Damiano E., Greco R., Minardo A., Olivares L., Zeni L. (2015). Performance of Slope Behavior Indicators in Unsaturated Pyroclastic Soils. J. Mt. Sci..

[30] Marjanović M., Caha J., Miřijovský J. (2015). Proposition of a Landslide Monitoring System in Czech Carpathians. Engineering Geology for Society and Territory.

[31] Barrias A., Casas J.R., Villalba S. (2016). A review of distributed optical fiber sensors for civil engineering applications. Sensors.

[32] Li M., Cheng W., Chen J., Xie R., Li X. (2017). A High Performance Piezoelectric Sensor for Dynamic Force Monitoring of Landslide. Sensors.

[33] Zhang S.J., Jiang C. (2017). An experimental study: Integration device of fiber bragg grating and reinforced concrete beam for measuring debris flow impact force. J. Mt. Sci..

[34] Yeo T.L., Sun T., Grattan K.T.V. (2008). Fibre-optic sensor technologies for humidity and moisture measurement. Sens. Actuators A Phys..

[35] Alwis L., Sun T., Grattan K.T.V. (2013). Optical fibre-based sensor technology for humidity and moisture measurement: Review of recent progress. Measurement.

[36] Pei H.F., Li C., Zhu H.H., Wang Y.J. (2013). Slope stability analysis based on measured strains along soil nails using FBG sensing technology. Math. Probl. Eng..

[37] Zhu H.H., Shi B., Yan J.F., Zhang J., Wang J. (2015). Investigation of the evolutionary process of a reinforced model slope using a fiber-optic monitoring network. Eng. Geol..

[38] Su Y.J., Xu H.Z., Gu P., Hu W.J. (2017). Application of FBG sensing technology in stability analysis of geogrid-reinforced slope. Sensors.

[39] Xu H., Zheng X., Zhao W., Sun X., Li F., Du Y., Liu B., Gao Y. (2019). High Precision, Small Size and Flexible FBG Strain Sensor for Slope Model Monitoring. Sensors.

[40] Gage J.R., Fratta D., Turner A.L., MacLaughlin M.M., Wang H.F. (2013). Validation and implementation of a new method for monitoring in situ strain and temperature in rock masses using fiber-optically instrumented rock strain and temperature strips. Int. J. Rock Mech. Min. Sci..

[41] Sun A., Semenova Y., Farrell G., Chen B., Li G., Lin Z. (2010). BOTDR integrated with FBG sensor array for distributed strain measurement. Electron. Lett..

[42] Huntley D., Bobrowsky P., Qing Z., Sladen W., Bunce C., Edwards T., Hendry M., Martin D., Choi E. (2014). Fiber Optic Strain Monitoring and Evaluation of a Slow-Moving Landslide Near Ashcroft, British Columbia, Canada. Landslide Science for a Safer Geoenvironment.

[43] Arslan A., Kelam M.A., Eker A.M., Akgün H., Koçkar M.K. (2015). Optical Fiber Technology to Monitor Slope Movement. Engineering Geology for Society and Territory.

[44] Dost B., Gronz O., Casper M., Krein A. (2020). The Potential of Smartstone Probes in Landslide Experiments: How to Read Motion Data. Nat. Hazards Earth Syst. Sci. Discuss..

[45] Dai F.C., Lee C.F. (2001). Terrain-based mapping of landslide susceptibility using a geographical information system: A case study. Can. Geotech. J..

[46] Dai F.C., Lee C.F. (2002). Landslide characteristics and slope instability modeling using GIS, Lantau Island, Hong Kong. Geomorphology.

[47] Ayalew L., Yamagishi H., Ugawa N. (2004). Landslide susceptibility mapping using GIS-based weighted linear combination, the case in Tsugawa area of Agano River, Niigata Prefecture, Japan. Landslides.

